# Long-term prognostic value of left atrial longitudinal strain in an elderly community-based cohort

**DOI:** 10.1007/s11357-022-00673-6

**Published:** 2022-12-09

**Authors:** Fjolla Zhubi Bakija, Zsolt Bagyura, Alexandra Fábián, Andrea Ferencz, Loretta Kiss, Orsolya Szenczi, Réka Vadas, Edit Dósa, Dat Tin Nguyen, Csaba Csobay-Novák, Ádám L. Jermendy, Zsolt Szelid, Pál Soós, Attila Kovács, Béla Merkely

**Affiliations:** 1 Heart and Vascular Center, Semmelweis University, Városmajor str. 68, Budapest, 1122 Hungary; 2 Clinic of Cardiology, University and Clinical Center of Kosovo, Prishtina, Kosovo; 3 2nd Department of Pediatrics, Semmelweis University, Budapest, Hungary

**Keywords:** Risk stratification, Mortality, Echocardiography, Global longitudinal strain, Diastolic dysfunction

## Abstract

Despite the well-known importance of left atrial (LA) mechanics in diastolic function, data are scarce regarding the prognostic power of LA longitudinal strain and its potential added value in the risk stratification of an elderly population. Accordingly, our aim was to determine the long-term prognostic importance of 2D speckle-tracking echocardiography-derived peak atrial longitudinal strain (PALS) in a community-based screening sample. Three hundred and fourteen volunteers were retrospectively identified from a population-based screening program (mean age 62 ± 11 years; 58% female) with a median follow-up of 9.5 years. All subjects who participated in the screening program underwent 2D echocardiography to measure left ventricular (LV) ejection fraction (EF), global longitudinal strain (GLS), and PALS, as well as low-dose cardiac CT to determine the Agatston score. The primary endpoint was all-cause mortality. Thirty-nine subjects (12.4%) met the primary endpoint. Subjects with adverse outcomes had significantly lower LV GLS (dead vs. alive; − 19.2 ± 4.3 vs. − 20.6 ± 3.5%, *p* < 0.05) and PALS (32.3 ± 12.0 vs. 41.8 ± 14.2%, *p* < 0.001), whereas LV EF did not show a difference between the two groups (51.1 ± 7.0 vs. 52.1 ± 6.2, %, *p* = NS). By multivariable Cox regression analysis, PALS was found to be a significant predictor of adverse outcomes independent of LV GLS, and Agatston and Framingham scores. In subjects with PALS values below the standard cut-off of 39%, the risk of all-cause mortality was almost 2.5 times higher (hazard ratio: 2.499 [95% confidence interval: 1.334–4.682], *p* < 0.05). Beyond the assessment of LV EF and LV GLS, PALS offers incremental value in cardiovascular risk stratification in a community-based elderly cohort. PALS was found to be a significant and independent predictor of long-term mortality among other classical cardiovascular risk estimators.

**Supplementary Information:**

The online version contains supplementary material available at 10.1007/s11357-022-00673-6.

## Introduction

Heart failure (HF) remains a significant global healthcare burden, as one of the leading causes of mortality and morbidity in the elderly. Therefore, the granular risk stratification of individuals at risk is becoming even more crucial [1]. Echocardiography is a cost-effective method to obtain a vast amount of information about cardiac structure and function. Nevertheless, the long-term prognostic value of echocardiographic parameters must be justified before it can be utilized as a screening and stratification tool in the general population. Conventional echocardiographic metrics generally lack this predictive power. Recently, new echocardiographic techniques, such as speckle tracking echocardiography (STE), have been developed and utilized in clinical routines to address this issue [2]. STE is easy to use, reproducible, and offers new insights into the mechanics of the myocardium [2].

Left ventricular (LV) global longitudinal strain (GLS) measured by STE has the ability to sensitively detect systolic dysfunction, even when the commonly used measure of LV ejection fraction (LVEF) is still in the normal range [3]. Recent studies demonstrated that reduced GLS values were associated with adverse clinical outcomes in patients with heart failure, and its prognostic value was superior compared with LVEF [3–5]. Moreover, the added predictive power of GLS was also present in patients after myocardial infarction, where the reduction of GLS was again related to poor cardiovascular outcomes and mortality [6]. Thus, as an early indicator of cardiac dysfunction, GLS may be used to identify those who are at a greater risk of cardiovascular morbidity and mortality in the general population. Indeed, recent studies reported that GLS could be useful in the general population for predicting cardiovascular adverse outcomes [7–9].

However, LV diastolic dysfunction often precedes systolic dysfunction having similar detrimental downstream consequences in the long term. Thus, the assessment of LV diastolic function along with left atrial (LA) function may offer more sensitive biomarkers to detect early dysfunction and predict long-term prognosis. STE can be applied to the LA as well, and — unlike conventional parameters — LA strain deteriorates progressively with the severity of the underlying diseases; thus, its measurement offers a practical and precise categorization of diastolic dysfunction [10, 11]. LA strain seems to predict the occurrence of atrial fibrillation and even provides incremental value for embolism risk assessment over CHA_2_DS_2_-VASc score [12–15]. Despite the well-known importance of LA mechanics in diastolic function evaluation, data are scarce regarding the long-term prognostic power of LA longitudinal strain and its potential added value on top of LV function indices. Such an evidence may add another layer to our knowledge about the importance of LA deformation that can ultimately support the widespread utilization of this approach.

Accordingly, our aim was to determine the long-term prognostic importance of STE-derived peak atrial longitudinal strain (PALS) in a community-based screening sample comprising of elderly individuals.

## Methods

### Study sample

The Budakalász Study is a cross-sectional voluntary screening program comprising adult population from the Central Hungarian region aiming to collect information on their health state and cardiovascular risk profile and to discover new cardiovascular risk factors [16]. Study procedures include questionnaires, noninvasive tests (anthropometry, echocardiography, carotid duplex scan, blood pressure measurement, ankle-brachial index), and venous blood sample collection for laboratory tests. Females over 40 years of age and males over 35 years of age were invited to participate in the cardiac computed tomography (CT) substudy. To allow a comprehensive cardiovascular risk assessment, the inclusion criteria of our current study were (1) availability of transthoracic echocardiography, (2) availability of carotid intima-media thickness (IMT) measurement, and (3) availability of Agatston score. Patients with inadequate image quality for LV and LA longitudinal strain calculation by speckle-tracking analysis (i.e., unavailable apical four-chamber view loops, poor visualization of more than one LV segment or the LA roof on apical four-chamber view, inadequate tracking by expert visual assessment or by the morphology of the segmental time-strain curves) were excluded.

All participants provided written informed consent to study procedures. Our study is in accordance with the Declaration of Helsinki and approved by the Medical Research Council (ETT-TUKEB No. 13687–0/2011-EKU).

### General medical examination

As a part of the medical examination, complete medical history was obtained, with special emphasis given to cardiovascular diseases, their signs, and symptoms, as well as lifestyle factors (alcohol consumption, smoking, physical activity), family history, and medications. Blood samples were taken for laboratory analysis. Body weight and height were measured; then, body mass index (BMI) and body surface area (BSA) were calculated. A 12-lead electrocardiogram (ECG) was also recorded [16]. Blood pressure was measured on a supine position on both arms after 20 min of rest using validated equipment. An individual was considered to have hypertension, hyperlipidemia, or diabetes mellitus if the disease was previously diagnosed or treated, based on medical history. Coronary artery disease was defined as history of myocardial infarction, previous coronary artery bypass grafting (CABG), or percutaneous coronary intervention (PCI). Using the Framingham risk score, we calculated the individual’s 10-year risk of manifesting clinical cardiovascular disease [17].

### Carotid duplex scan

A duplex scan of both carotid arteries was performed (Vivid i ultrasound system, 12L-RS linear probe, GE Healthcare, Horten, Norway), which was postprocessed offline (GE EchoPAC) to measure carotid IMT according to the recommendations of the 2012 Mannheim Consensus. Additionally, a detailed description of the carotid plaques (if any) was performed [18].

### Cardiac CT

Females over 40 years of age and males over 35 years of age were offered to have a cardiac CT scan, which was performed on a 256-slice CT machine (Brilliance iCT, Philips Healthcare, Best, The Netherlands) and were exposed to a radiation dose of 0.5 mSv or less. Quantitative analysis of coronary calcification on the axial images was performed using a commercially available software (Calcium scoring, Heartbeat-CS, Philips), and the Agatston score was calculated and reported as 0 and non-0 for each patient [19].

### Echocardiographic assessment

Using commercially available ultrasound system (Vivid i, 3Sc-RS transducer), three experienced echocardiographers obtained all echocardiograms. All participants underwent a standard focused protocol comprising two-dimensional (2D) imaging and tissue Doppler imaging (TDI). The acquired images were analyzed offline by two experienced investigators blinded to outcomes and clinical data using commercially available software package (GE EchoPAC).

#### Conventional echocardiography

LV internal diameters, wall thicknesses, and relative wall thickness were measured using the 2D-guided linear method. End-diastolic LV dimensions were used to calculate LV mass by an anatomically validated formula according to the recommendations of the American Society of Echocardiography [2]. We determined LV end-diastolic volume (EDV), end-systolic volume (ESV), and their BSA-indexed values along with LVEF using the monoplane or the biplane Simpson’s method. Left atrial end-systolic volume (LAV) was measured using the Simpson's method in the apical four-chamber view and was indexed to BSA to calculate LAVi [2]. Mitral inflow was assessed using pulsed-wave Doppler between the tips of the mitral leaflets in the apical four chamber view. Peak early (E) and late (A) diastolic inflow velocities were measured and used to determine the E/A ratio. Furthermore, the deceleration time (DT) of the *E*-wave was measured. From the TDI recordings, we measured systolic (*s*′), early diastolic (*e*′), and late diastolic (*a*′) velocities of the mitral lateral and septal annulus. We calculated the *E*/*e*′ ratio by dividing trans-mitral *E* velocity by *e*′ averaged from these sites. Concerning the right heart, right ventricular basal short-axis diameter (RVd) and tricuspid annular plane systolic excursion (TAPSE) were also measured. Right atrial end-systolic volume (RAV) was measured using the Simpson’s method in the apical four-chamber view and was indexed to BSA to calculate RAVi.

#### Speckle-tracking echocardiography

We used a dedicated, vendor-independent speckle-tracking software package (AutoStrain LV and AutoStrain LA, TomTec Imaging Systems, Unterschleißheim, Germany) to analyze LV and LA deformation using the apical four-chamber view. To avoid further patient dropout due to image quality, no attempt was made to analyze apical two-chamber and long-axis views. The primary measurements of interest included the LV GLS (average of six LV segments) and peak atrial longitudinal strain (PALS) referring to the reservoir function of the LA [20, 21].

In brief, these software automatically identify apical four-chamber view along with LV and LA endocardial border and track the longitudinal deformation frame-by-frame throughout the cardiac cycle. In case of suboptimal ECG or 2D echocardiographic image quality, correction of cardiac cycle events or the endocardial contour may be necessary. The segmental peak negative values (longitudinal shortening) were used to calculate LV GLS. The peak positive value of the LA strain curve (lengthening in systole — reservoir function) was used to calculate PALS, and the late diastolic peak or plateau before *P* wave was used to calculate peak atrial contraction strain (PACS). The ventricular end-diastole was used as the reference time point for LA strain analysis. For PALS, we used a conventional cut-off of 39% [22].

### Study outcomes

Follow-up data (status [dead or alive], date of death) was obtained from Hungary’s National Health Insurance Database. The primary endpoint of our study was all-cause mortality.

### Statistical analysis

Statistical analysis was performed using a dedicated software (SPSS v22, IBM, Armonk, NY, USA). Continuous variables are expressed as mean ± standard deviation (SD), whereas categorical variables were reported as frequencies and percentages. After the verification of normal distribution of variables using the Shapiro–Wilk test, the clinical and echocardiographic characteristics were compared with unpaired Student’s *t* test or Mann–Whitney *U* test for continuous variables, and chi-squared or Fisher’s exact test for categorical variables, as appropriate. Cox proportional hazards models were used to compute hazard ratios (HRs) with 95% confidence intervals (95% CIs). Including significant variables identified at the univariable Cox regression analysis, multivariable Cox regression models were built to identify independent predictors of outcomes. Collinearity of variables was tested at each multivariable model by variance inflation factor (excessive if variance inflation factor > 3). Receiver-operator characteristic (ROC) curves were generated to assess the discriminatory power of PALS with regard to the endpoint. Youden’s index was used to identify the optimal cut-off point; then, this value or the conventionally used 39% value was used to dichotomize the study population. Outcomes of the dichotomized groups were visualized on Kaplan–Meier curves and compared by log-rank test. A two-sided *P*-value of 0.05 was considered statistically significant.

## Results

### Baseline demographic and clinical characteristics

Three hundred and fourteen individuals were retrospectively identified with previous 2D transthoracic echocardiographic examination available. During a median follow-up of 9.5 [interquartile range: 9.1–9.9] years, 39 subjects met the primary endpoint of all-cause mortality.

Baseline demographic and clinical characteristics are summarized in Table 1. Subjects with adverse outcomes were significantly older, whereas there was no difference in sex within the study groups, although the overall study population showed a slight female predominance. The BMI values of subjects with adverse outcomes were significantly higher, while the BSA values remained similar in the two groups. Subjects who experienced adverse outcomes had higher systolic blood pressures; however, there was no difference in diastolic blood pressure and heart rate in the study groups. Concerning cardiovascular risk factors, 58% of study subjects (183 subjects) had a history of hypertension, which was the most prevalent cardiovascular risk factor among the study population and was less frequent among the subjects who met the endpoint (46.2% vs. 60%). Furthermore, 17.5% of the study population was diagnosed with diabetes mellitus. In the overall study population, 130 persons (41.4%) were reported as smokers, and the frequency of smoking was significantly higher in subjects with adverse outcome. Among the study cohort, 12.7% had a history of arrhythmias, whereas 4.5% had a previous stroke, and 6.7% had pulmonary disease in medical history. Coronary artery disease was represented by the number of subjects who had a history of myocardial infarction (MI, 13 persons) or underwent previous PCI or previous CABG procedures in the overall study population (PCI: 3 persons, 0.9%; CABG: 1 person, 0.3%) (Table 1).

**Table 1:** Demographic and clinical characteristics

	Overall (*n* = 314)	Alive (*n* = 275)	Deceased (*n* = 39)	*p*-value
Baseline demographic characteristics				
Age, years	61.5 ± 10.7	60.5 ± 10.3	68.6 ± 10.8	< 0.001
Females	182 (57.9)	165 (60.0)	17 (43.6)	0.052
Clinical characteristics				
BSA, m^2^	1.89 ± 0.22	1.88 ± 0.22	1.95 ± 0.18	0.064
BMI, kg/m^2^	28.5 ± 4.9	28.1 ± 4.9	30.9 ± 4.7	< 0.001
Systolic blood pressure, mmHg	137.9 ± 18.9	136.8 ± 18.6	145.7 ± 19.4	0.006
Diastolic blood pressure, mmHg	78.9 ± 11.0	79.1 ± 11.0	78.3 ± 10.9	0.679
Heart rate, bpm	69.2 ± 10.3	68.9 ± 9.9	71.4 ± 12.3	0.153
Risk factors and medical history				
Smoking history	130 (41.4)	108 (39.2)	22 (56.4)	0.042
Hypertension	183 (58.0)	165 (60.0)	18 (46.2)	0.101
Diabetes mellitus	55 (17.5)	49 (17.8)	6 (15.4)	0.708
Arrhythmia	40 (12.7)	33 (12.0)	7 (17.9)	0.297
Previous MI	13 (4.1)	13 (4.7)	0 (0)	0.165
Previous PCI	3 (0.9)	3 (1.1)	0 (0)	0.512
Previous CABG	1 (0.3)	1 (0.4)	0 (0)	0.706
Previous stroke	14 (4.5)	13 (4.7)	1 (2.6)	0.540
Pulmonary disease	21 (6.7)	17 (6.2)	4 (10.3)	0.194
Framingham risk score	19.4 ± 12.3	17.9 ± 11.5	29.9 ± 12.6	< 0.001
Agatston score (non-0)	204 (64.9)	170 (61.8)	34 (87.2)	0.002
Carotid IMT, mm	0.73 ± 0.14	0.71 ± 0.14	0.80 ± 0.12	0.001
Laboratory parameters				
Total cholesterol, mmol/L	5.7 ± 1.2	5.7 ± 1.1	5.3 ± 1.3	0.027
HDL-cholesterol, mmol/L	1.5 ± 0.5	1.5 ± 0.5	1.5 ± 0.5	0.575
LDL-cholesterol, mmol/L	3.5 ± 1.0	3.6 ± 0.9	3.2 ± 1.3	0.030
Triglycerides, mmol/L	2.3 ± 1.4	2.3 ± 1.4	2.2 ± 1.4	0.737
Glucose, mmol/L	6.2 ± 1.9	6.1 ± 1.7	6.6 ± 2.5	0.140
ProBNP, pmol/L	127.2 ± 181.9	104.1 ± 114.5	293.0 ± 385.6	< 0.001
Serum creatinine, µmol/L	77.2 ± 16.8	76.5 ± 16.2	82.2 ± 19.7	0.045
eGFR, ml/min	82.1 ± 16.4	83.0 ± 15.9	75.8 ± 18.5	0.011
HbA1c, %	5.9 ± 0.8	5.8 ± 0.7	6.3 ± 1.2	0.001

Continuous variables are presented as means ± SD; categorical variables are reported as *n* (%)

*BMI* body mass index, *BSA* body surface area, *CABG* coronary artery bypass grafting, *eGFR* estimated glomerular filtration ratio, *HDL* high-density lipoprotein, *IMT* intima-media thickness, *LDL* low-density lipoprotein, *MI* myocardial infarction, *PCI* percutaneous coronary intervention

Regarding laboratory parameters, total cholesterol, low density lipoprotein (LDL)-cholesterol, and estimated glomerular filtration rate (eGFR) levels were significantly lower, whereas pro hormone B-type natriuretic peptide (ProBNP), serum creatinine, and hemoglobin A1c (HbA1c) levels were significantly higher among subjects who met the endpoint. On the other hand, high-density lipoprotein (HDL)-cholesterol, triglycerides, and serum glucose levels did not show a difference between the study groups (Table 1).

### Conventional 2D and speckle-tracking echocardiographic parameters

Conventional 2D echocardiographic parameters of the study population are shown in Table 2. LV end-diastolic internal diameter and calculated LV mass index (LV Mi) values were significantly higher in subjects with adverse outcome, in contrast to wall thicknesses and relative wall thickness (RWT) values, which did not differ between the two groups. Subjects who experienced adverse outcomes showed significantly higher values of indexed LV end-systolic volume (LV ESVi) and indexed end-diastolic volume (LV EDVi). LV EF, on the other hand, did not show a difference between study groups. Concerning diastolic function, subjects who met the endpoint demonstrated significantly higher transmitral *A*-wave velocity and lower *E*/*A* ratios along with a significantly longer deceleration time. Transmitral *E*-wave velocity, on the other hand, did not show a difference. Furthermore, mitral annular early diastolic velocities were significantly lower, whereas the average *E*/*e*′ ratio was higher in subjects with adverse outcome. 2D LA volume index was significantly higher among subjects who met the endpoint. Regarding the right heart, RV basal diameter did not show any difference, similarly to TAPSE and 2D RA volume index which also did not differ between the two study groups (Table 2).

**Table 2:** Echocardiographic parameters of the study population

	Overall (*n* = 314)	Alive (*n* = 275)	Deceased (*n* = 39)	*p*-value
2D conventional echocardiographic data				
LVIDd, mm	47.9 ± 4.7	47.7 ± 4.5	50.1 ± 5.9	0.003
IVSd, mm	9.9 ± 1.8	9.9 ± 1.8	10.3 ± 1.9	0.221
PWd, mm	9.7 ± 1.6	9.6 ± 1.6	10.1 ± 1.6	0.078
RWT, %	0.41 ± 0.08	0.41 ± 0.07	0.41 ± 0.09	0.636
LV Mi, g/m^2^	88.7 ± 22.8	87.7 ± 22.7	96.0 ± 23.0	0.042
LV ESVi, ml/m^2^	38.6 ± 13.1	37.9 ± 12.1	43.3 ± 17.7	0.018
LV EDVi, ml/m^2^	79.9 ± 22.1	78.9 ± 21.2	87.5 ± 26.3	0.021
LV EF, %	51.9 ± 6.3	52.1 ± 6.2	51.1 ± 7.0	0.347
* E*, cm/s	75.7 ± 18.9	75.3 ± 16.7	78.3 ± 30.2	0.358
* A*, cm/s	75.5 ± 22.9	74.1 ± 22.5	86.2 ± 22.3	0.002
* E*/*A*	1.09 ± 0.48	1.11 ± 0.47	0.94 ± 0.49	0.039
DT (ms)	211.9 ± 62.7	208.8 ± 59.9	234.7 ± 77.0	0.017
Mitral lateral *s*′, cm/s	8.9 ± 2.5	8.9 ± 2.4	8.6 ± 3.0	0.567
Mitral lateral *e*′, cm/s	10.3 ± 3.3	10.5 ± 3.3	8.7 ± 2.2	0.002
Mitral lateral *a*′, cm/s	10.7 ± 3.2	10.7 ± 3.1	11.1 ± 4.3	0.463
Mitral medial *s*′, cm/s	8.36 ± 1.9	8.39 ± 1.8	8.1 ± 1.9	0.427
Mitral medial *e*′, cm/s	8.8 ± 2.9	9.1 ± 2.9	7.3 ± 2.5	< 0.001
Mitral medial *a*′, cm/s	11.0 ± 2.7	11.01 ± 2.6	10.9 ± 3.2	0.884
* E*/*e*′ average	8.4 ± 3.1	8.11 ± 2.9	10.0 ± 3.9	< 0.001
LAVi, ml/m^2^	33.2 ± 10.4	32.3 ± 9.7	38.9 ± 12.7	< 0.001
RVd, mm	35.2 ± 5.8	35.1 ± 5.8	35.6 ± 5.5	0.623
TAPSE, mm	24.7 ± 4.2	24.7 ± 4.0	24.3 ± 5.0	0.595
RAVi, ml/m^2^	22.5 ± 11.6	22.1 ± 11.6	25.7 ± 11.2	0.069
Speckle tracking echocardiography data				
LV GLS, %	20.5 ± 3.7	20.6 ± 3.5	19.2 ± 4.3	0.022
PALS, %	40.6 ± 14.2	41.8 ± 14.2	32.3 ± 11.9	< 0.001
PACS, %	18.8 ± 8.7	18.9 ± 8.8	17.2 ± 7.9	0.225

Continuous variables are presented as means ± SD

*A* atrial contraction, *a′* peak late (atrial) diastolic annular velocity, *DT* deceleration time, *E* early diastolic filling, *e′* early diastolic annular velocity, *EDV* end diastolic volume index, *EF* ejection fraction, *ESVi* end-systolic volume index, *IVSd* inter-ventricular septal diameter, *LAVi* left atrial volume index, *LV* left ventricle, *LV GLS* left ventricular global longitudinal strain, *LVIDd* left ventricular internal diameter at end-diastole, *LV Mi* left ventricular mass index, *PWd* posterior wall diameter, *PACS* peak atrial contraction strain, *PALS* peak atrial longitudinal strain, *RAVi* right atrial volume index, *RVd* right ventricle diameter, *RWT* relative wall thickness, *s′* systolic annular velocity, *TAPSE* tricuspid annular plane systolic excursion

We have compared the subjects with and without adverse outcomes based on speckle-tracking echocardiography-derived data. The results are shown in Table 2. As expected, there were significant differences between the two groups regarding LV and LA strain parameters. In subjects with adverse outcome, LV GLS showed decreased values compared to those without. Concerning LA mechanics, PALS showed significantly lower values in subjects who met the endpoint. Conversely, peak atrial contraction strain (PACS) did not show a difference between the two study groups (Table 2).

### Prognostic value and discriminatory power of PALS

Including significant variables identified at the univariable Cox regression analysis (Supplementary Table 1), multivariable Cox regression analysis were performed and the results are summarized in Table 3. In order to identify independent predictors of outcomes and to determine the prognostic value of PALS, three multivariable Cox regression models were built, including Framingham risk score and PALS in each model. In Model 1, comprising Framingham risk score, PALS, and LV GLS, PALS (HR: 0.967 [95% CI: 0.939–0.995], *p* < 0.05) was found to be independently associated with the adverse outcome along with Framingham risk score. In Model 2, consisting of Framingham risk score, PALS, and carotid IMT, all three variables were independently associated with all-cause mortality (PALS HR: 0.954 [95% CI: 0.924–0.985], *p* < 0.05). Concerning Model 3 which comprised Framingham risk score, PALS, and Agatston score, only PALS (HR: 0.967 [95% CI: 0.941–0.993], *p* < 0.05) and Framingham risk score were found to be independently associated with the adverse outcome (Table 3).

**Table 3:** Independent predictors of all-cause mortality identified using multivariable Cox regression

	Multivariable Cox regression					
	Model 1		Model 2		Model 3	
	HR [95% CI]	*p*-value	HR [95% CI]	*p*-value	HR [95% CI]	*p*-value
Framingham risk score	1.056 [1.032–1.081]	< 0.001	1.042 [1.015–1.071]	0.003	1.046 [1.020–1.073]	< 0.001
PALS	0.967 [0.939–0.995]	0.023	0.954 [0.924–0.985]	0.004	0.967 [0.941–0.993]	0.012
LV GLS	1.032 [0.938–1.134]	0.521	-		-	
Carotid IMT	-		14.619 [1.036–206.238]	0.047	-	
Agatston score	-		-		2.536 [0.742–8.669]	0.138

*CI* confidence interval, *HR* hazard ratio, *IMT* intima-media thickness, *LV GLS* left ventricular global longitudinal strain, *PALS* peak atrial longitudinal strain

**Fig. 1:**
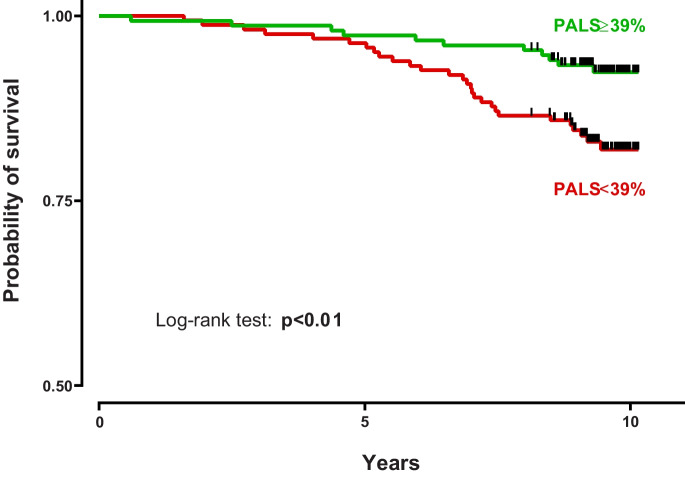
Kaplan–Meier survival curves using a standard cut-off value of 39%

Using the conventional cut-off value of 39%, PALS was able to discriminate between a high-risk and a low-risk group in terms of all-cause mortality. As depicted by Kaplan–Meier curves, in subjects with lower PALS values, the risk of all-cause mortality was almost 2.5 times higher than in subjects with PALS values above 39% (HR: 2.499 [95% CI: 1.334–4.682], *p* < 0.05) (Fig. 1).

**Fig. 2:**
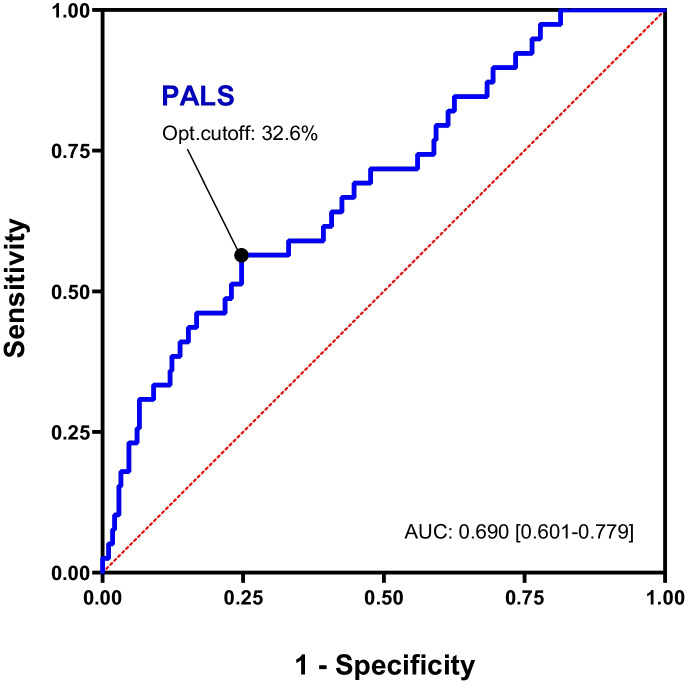
Receiver operating characteristic curve illustrating the discriminatory power of PALS with regard to the endpoint. Youden’s index was used to identify the optimal cut-off point of 32.6%

**Fig. 3:**
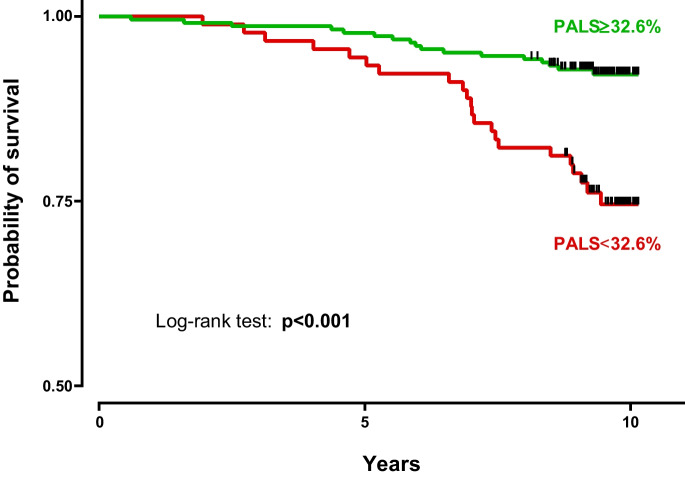
Kaplan–Meier survival curves based on the optimal cut-off value (32.6%) of PALS assessed with receiver operating characteristic analysis

We also performed ROC analysis to assess the discriminatory power of PALS with regard to the endpoint. The area under the ROC curve (AUC: 0.690 [95% CI: 0.601–0.779) is shown in Fig. 2. Using Youden’s index, we calculated the optimal cut-off value of 32.6% with a sensitivity of 56.4% and specificity of 75.3% (Fig. 2). When using this cut-off, in subjects with lower PALS values, the risk of all-cause mortality was more than three times higher than in subjects with PALS values above 32.6% (HR: 3.424 [95% CI: 1.694–6.919], *p* < 0.001) (Fig. 3).

## Discussion

**Fig. 4:**
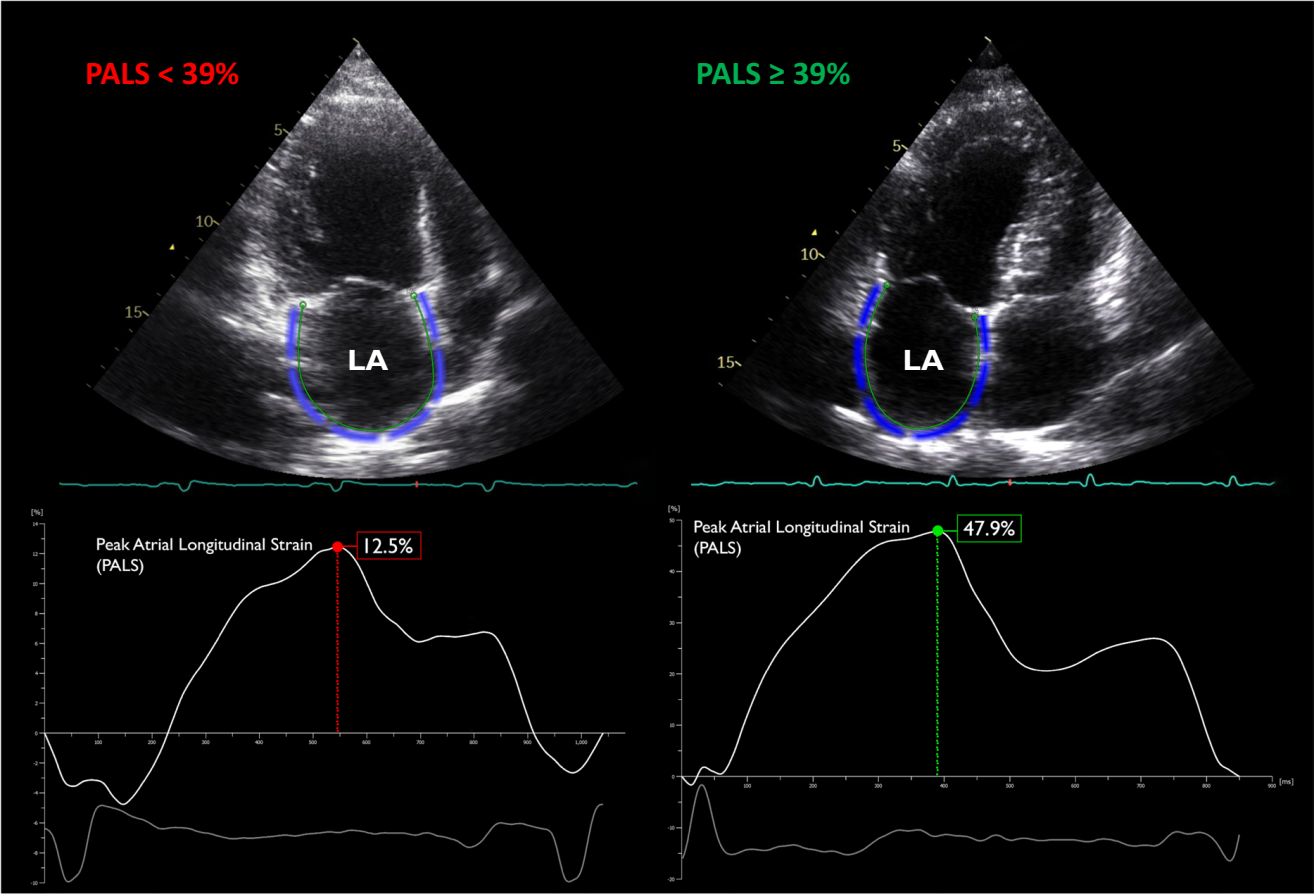
Representative cases of subjects below (indicated with red) and above 39% (indicated with green) of peak atrial longitudinal strain (PALS). Subject with 12.6% PALS met the primary endpoint during the follow-up period. The blue contour on the 2D echocardiographic image depicts left atrial (LA) endocardial border at end-systole

In this study, we assessed the utility of PALS in predicting long-term all-cause mortality in an elderly, community-based screening sample. We demonstrated that PALS was a strong and independent predictor of all-cause mortality in the community and offered incremental value over LV functional metrics including GLS. Furthermore, by dichotomizing the population based on the standard cut-off PALS value of 39% [22], we have also showed that in subjects with abnormal PALS values, the risk of all-cause mortality was almost 2.5 times higher (Fig. 4).

In the era of the increasing recognition of HF with preserved LV EF (HFpEF), clinical and research efforts have turned the spotlight back on diastolic dysfunction. Beyond the conventional echocardiographic parameters of diastolic function (mitral inflow velocities, TDI-derived metrics, etc.), the assessment of LA mechanics is of particular interest. Novel techniques enable the automated and accurate measurement of all three LA phasic functions, i.e., the reservoir (filling during ventricular systole), the conduit (passive emptying during early diastole), and the contraction (active contraction in late diastole). Reservoir strain (PALS) by 2D speckle tracking echocardiography has emerged as the most powerful parameter. In our study, we have proved that PALS is a strong predictor of long-term outcomes in a community-based sample. Importantly, the predictive value of PALS was independent of LV GLS, carotid IMT, Agatston score, and also the Framingham risk score. These results hold the potential to introduce LA mechanical assessment into routine screening protocols among the elderly, whose echocardiographic evaluation and risk stratification is a challenge in routine clinical practice.

LV diastolic function normally deteriorates with age [23–25]. Still, there is evidence that LV diastolic dysfunction and increased LA volumes are independent predictors of first age-related cardiovascular event in an elderly population [26]. Cacciapuoti et al. also confirmed the connection between LA function and volumes and age-related LV dysfunction [23]. With regard to conventional echocardiographic parameters of the LA, numerous studies presented their diagnostic and prognostic role in various clinical scenarios. The most commonly used parameter to assess left atrial structure is LAVi. Over 3.5 years of follow-up of 317 patients enrolled in sinus rhythm, LAVi outperformed LA area and M-mode LA dimensions in predicting the composite endpoint of the first occurrence of atrial fibrillation (AF), congestive HF, stroke, transient ischemic attack, acute myocardial infarction (AMI), coronary revascularization, and cardiovascular mortality. In addition, LAVi showed a graded association with overall event-free survival [27]. A recent study by Inciardi et al. involving 4901 elderly participants confirmed that regardless of measures of LV function and NT-proBNP, higher minimal LAVi values, but not maximal LAVi values, along with novel indices of LA function were associated with a greater risk of incident HF and death [26].

Nevertheless, even a normal-size LA can be dysfunctional, and LAVi has been shown to have low sensitivity in the early detection of LV diastolic dysfunction [28]. Two-dimensional STE, on the other hand, is a readily available, semi-automated, and reproducible method that allows direct measurements of LA mechanics. Several recent investigations have shown that PALS is a reliable indicator of LA reservoir function and is closely connected to LV filling pressures [29, 30]. In HFpEF, a recent study suggested that PALS has an important clinical and prognostic relevance, underpinning the active role of the LA in the pathophysiology of the disease course. PALS is consistently linked to the severity of diastolic dysfunction [11]. After analyzing a population of 517 patients at risk for LV diastolic dysfunction, Morris et al. found that adding PALS to LAVi increased the detection rate of LV diastolic dysfunction. In terms of clinical significance, even when LAVi was in a normal range, the deterioration of PALS was strongly associated with worsening New York Heart Association functional class [28].

With regard to the risk assessment in the general population, some recent studies have shown the predictive value of other STE-derived parameters such as LV GLS [7–9]. In a large community-based cohort, low LV GLS predicted future cardiovascular events independent of conventional risk factors after a follow-up of 7.9 years. Authors revealed that the risk for cardiovascular events increased with increasing number of left ventricular abnormalities, such as low LV GLS, diastolic dysfunction, and LV hypertrophy [7]. In another study, LV GLS provided incremental prognostic information beyond the Framingham risk score, the SCORE risk chart, and the modified ACC/AHA Pooled Cohort Equation for the composite outcomes and incident HF, after analyzing 1296 low-risk subjects. Of note, GLS was an independent predictor of outcomes in men but not in women [8]. In our cohort, GLS was indeed impaired in the patient group with adverse outcome despite similar LVEF; however, in the multivariable model PALS emerged as the independent predictor of outcome beyond the Framingham risk score. We may hypothesize that LA functional deterioration (diastolic dysfunction) precedes the development of LV systolic dysfunction, and thus, assessment of LA mechanics may allow an earlier detection of cardiac abnormalities turning into better predictive power for future adverse events.

Previously, a similar study to ours was conducted by Modin et al. [31], where the authors evaluated the prognostic value of PALS in the general population. They included 385 low-risk participants, and after the median follow-up of 12.6 years, 51 participants reached the composite endpoint. They have found that PALS was a univariable predictor of adverse outcomes. However, its predictive value was modified by sex as it was not a significant predictor in men [31]. Another study tested the role of PALS in predicting cardiovascular outcomes in a prospective cohort of 312 adults [32]. The authors concluded that PALS is a strong and independent predictor of cardiovascular adverse events and appears to be superior to other conventional echocardiographic parameters of the LA**.** Furthermore, Cameli et al. also performed ROC analysis, and among all LA parameters, PALS showed the highest discriminatory power to identify patients-at-risk in an older (71 ± 6 years) and non-low risk population. However, this study did not demonstrate whether the prognostic value of PALS was independent from LV function and was also limited by its relatively short follow-up (3.1 ± 1.4 years) [32]. Compared to the above-mentioned studies, our results add another layer of clinical significance showing that PALS is a significant predictor of long-term adverse outcomes and has independent value from other “classical” cardiovascular risk estimators such as LV function, IMT, and the Agatston and Framingham scores.

Our findings suggest that the measurement of PALS by STE could be beneficial in the risk stratification of the general population in a convenient and cost-effective manner. This could also aid in the early detection of those at risk for future adverse outcomes and identifying the best candidates for long-term preventive measures. Because even a minor reduction in risk factor levels results in a significant reduction in incident rates, the potential for cardiac disease prevention, especially in older individuals is considerable. Our results do not diminish, rather reinforce the value of the other imaging biomarkers investigated in the framework of this study. The Agatston score, along with IMT, is an established cumulative marker of cardiovascular damage having important prognostic value even in asymptomatic patients or during directly non-cardiac illnesses [33].

### Strengths and limitations

A crucial strength of the current study is the community-based design with a balanced distribution of male and female participants along with a relatively long-term follow-up. Furthermore, the subjects were examined with advanced imaging modalities. More importantly, our findings were confirmed in multivariable models.

Several limitations of this study merit consideration. The relatively small sample size with 314 participants is the first to mention. Secondly, we observed the association between PALS and all-cause mortality, without defining a specific cause of mortality. In addition, the extent of our multivariable analysis was also limited by the small number of events and also the inherent shortcomings of such models and their evaluation. Strain analysis was performed by tracing the LA endocardium in only one imaging plane (apical four-chamber view). However, recent guidelines recommend calculating PALS obtained from a single apical four-chamber view [20]. Additional studies, in larger cohorts with specified outcomes and adequate event rates, are needed to confirm our results. Using ROC analysis, PALS was presented with a modest sensitivity suggesting that it is not an optimal method for screening purposes on its own. However, its potential clinical use is to rather add it on top of a conventional echocardiographic protocol, as this way it can support clinical decision making in a powerful manner. Of note, deformation imaging overall is still underutilized in clinical routine that hinders the direct translation of our results to practice. However, next generation, automated tools that allow the quick and reproducible measurement of PALS may pave the way for the widespread utilization of this clinically meaningful metric.

## Conclusions

Beyond the assessment of LV systolic functional parameters such as LV EF and GLS, LA mechanics, namely, PALS, offers incremental value in cardiovascular risk stratification in a community-based cohort. By multivariable regression models, PALS was found to be a significant and independent predictor of long-term all-cause mortality. These results emphasize the importance of a thorough evaluation of LA mechanics in an elderly population.

## Supplementary Information

Below is the link to the electronic supplementary material.

Supplementary file1 (DOCX 17.9 KB)
