# Supplementary material for: Long-term prognostic value of left atrial longitudinal strain in an elderly community-based cohort
Source: GeroScience. 2022 Dec 9;45(1):613–25. doi: 10.1007/s11357-022-00673-6 (PMC9886757; doi:10.1007/s11357-022-00673-6)
Supplement: Supplementary file 1 — Supplementary file1 (DOCX 17.9 KB) [file 11357_2022_673_MOESM1_ESM.docx]

**Supplementary Table 1. Factors associated with all-cause mortality using univariable Cox regression**

| **Univariable Cox regression** | | |
| --- | --- | --- |
|  | **HR [95% CI]** | **p-value** |
| Age | 1.087 [1.049 - 1.127] | <0.001 |
| Framingham risk score | 1.065 [1.041 - 1.089] | <0.001 |
| Agatston score (non 0) | 5.302 [1.628 - 17.264] | 0.006 |
| Carotid IMT | 47.287 [5.210 - 429.163] | <0.001 |
| LVIDd | 1.093 [1.032 - 1.158] | 0.003 |
| IVSd | 1.109 [0.946 - 1.300] | 0.202 |
| PWd | 1.175 [0.990 - 1.394] | 0.065 |
| RWT | 2.871 [0.050 - 165.706] | 0.610 |
| LV Mi | 1.013 [1.001 - 1.026] | 0.034 |
| LV ESVi | 1.028 [1.007 - 1.049] | 0.009 |
| LV EDVi | 1.021 [1.004 - 1.038] | 0.014 |
| LV EF | 0.975 [0.927 - 1.026] | 0.333 |
| E | 1.006 [0.990 - 1.022] | 0.450 |
| A | 1.018 [1.007 - 1.029] | 0.002 |
| E/A | 0.330 [0.119 - 0.916] | 0.033 |
| DT | 1.005 [1.001 - 1.009] | 0.017 |
| Mitral lateral s*'* | 0.960 [0.837 - 1.100] | 0.554 |
| Mitral lateral e*'* | 0.837 [0.748 - 0.936] | 0.002 |
| Mitral lateral a*'* | 1.039 [0.940 - 1.147] | 0.456 |
| Mitral medial s*'* | 0.930 [0.773 - 1.119] | 0.444 |
| Mitral medial e' | 0.799 [0.697 - 0.915] | 0.001 |
| Mitral medial a*'* | 0.996 [0.881 - 1.126] | 0.948 |
| E/e*'* average | 1.150 [1.062 - 1.245] | <0.001 |
| LAVi | 1.047 [1.021 - 1.073] | <0.001 |
| RVd | 1.014 [0.960 - 1.071] | 0.611 |
| TAPSE | 0.820 [0.364 - 1.846] | 0.631 |
| RAVi | 1.024 [0.998 - 1.051] | 0.073 |
| LV GLS | 1.110 [1.017 - 1.211] | 0.020 |
| PALS | 0.949 [0.924 - 0.974] | <0.001 |
| PACS | 1.022 [0.986 - 1.060] | 0.234 |

*A, atrial contraction; a’, peak late (atrial) diastolic annular velocity; CI, confidence interval; DT, deceleration time; E, early diastolic filling; e’, early diastolic annular velocity; EDVi, end diastolic volume index; EF, ejection fraction; ESVi, end-systolic volume index; HR, hazard ratio; IMT, intima-media thickness; IVSd, inter-ventricular septal diameter; LAVi, left atrial volume index; LV, left ventricle; LV GLS, left ventricular global longitudinal strain; LVIDd, left ventricular internal diameter at end-diastole; LV Mi, left ventricular mass index; PWd, posterior wall diameter; PACS, peak atrial contraction strain; PALS, peak atrial longitudinal strain; RAVi, right atrial volume index; RVd, right ventricle diameter; RWT, relative wall thickness; s’, systolic annular velocity; TAPSE, tricuspid annular plane systolic excursion*
